# Molecular characterization and functional analysis of phagocytosis by human embryonic stem cell-derived RPE cells using a novel human retinal assay

**Published:** 2009-02-06

**Authors:** Amanda-Jayne Carr, Anthony Vugler, Jean Lawrence, Li Li Chen, Ahmed Ahmado, Fred K. Chen, Ma’ayan Semo, Carlos Gias, Lyndon da Cruz, Harry D. Moore, James Walsh, Peter J. Coffey

**Affiliations:** 1 Institute of Ophthalmology, University College London, London, UK; 2 Moorfields Eye Hospital, London, UK; 3 Department of Biomedical Science, Centre for Stem Cell Biology, University of Sheffield, UK; 4 Axordia Ltd, Sheffield, UK

## Abstract

**Purpose:**

To examine the ability of retinal pigment epithelial (RPE) cells derived from human embryonic stem cells (HESC) to phagocytose photoreceptor outer segments, and to determine whether exposure to human retina induces any morphological changes in these cells.

**Methods:**

HESC-RPE cells were derived from a super-confluent preparation of the Shef1 HESC line. Pigmented colonies were isolated and expanded into pigmented monolayers on Matrigel™ matrix-coated dishes or filters. Cells were exposed to fluorescently labeled outer segments isolated from the porcine eye and assessed for phagocytic activity at regular intervals. Expression of molecules associated with RPE phagocytosis was analyzed by RT–PCR, immunocytochemistry, and western blot. The role of Mer Tyrosine Kinase (MERTK) in the phagocytosis of outer segments was investigated using antibodies directed against MERTK to block function. In a novel approach, cells were also exposed to fresh human neural retina tissue then examined by electron microscopy for evidence of phagocytosis and changes in cell morphology.

**Results:**

HESC-derived RPE cells are capable of phagocytosing isolated porcine outer segments and express molecules associated with RPE-specific phagocytosis, including MERTK. Pre-incubation with antibodies against MERTK blocked phagocytosis of photoreceptor outer segments, but not polystyrene beads. HESC-RPE cells also phagocytosed outer segments in a novel human retinal explant system. Furthermore co-culture adjacent to human retina tissue in this preparation resulted in the appearance of features in HESC-derived RPE cells normally observed only as the RPE matures.

**Conclusions:**

The ingestion of photoreceptor outer segments from an isolated population and an artificial ex vivo human retina system demonstrates HESC-derived RPE cells are functional. HESC-derived RPE possess the relevant molecules required for phagocytosis, including MERTK, which is essential for the phagocytosis of outer segments but not latex beads. Furthermore, some changes observed in cell morphology after co-culture with human retina may have implications for understanding the full development and differentiation of RPE cells.

## Introduction

Age-related macular degeneration (AMD) is the leading cause of blindness in people over 60 in the western world, and as such, is a target for therapeutic intervention. The disease is associated with the progressive degeneration of the retinal pigment epithelium (RPE), leading to photoreceptor cell death and the loss of central vision. A potential cure for AMD could involve cell-based transplantation therapies to restore RPE cells lost during the progression of the disease. This could be achieved using RPE cells derived from human embryonic stem cells (HESC) as a replacement source. There is increasing evidence to suggest that HESC-derived RPE cells are more akin to RPE cells than cell lines originally created from human RPE tissue, when characterized in terms of morphology, gene expression, and immunohistochemical profile [1-4].

In vivo, the RPE constitutes a distinct monolayer of pigmented cells lying between the neural retina and Bruch’s membrane, which provides essential support for the long-term preservation of retinal integrity and visual function. RPE cells are involved in many processes critical for photoreceptor survival, including nutrient and ion transport, light absorption, recycling of retina, and formation of the blood-retinal barrier [5]. One of the most important functions of the RPE is the phagocytosis of photoreceptor outer segments (POS). Each day RPE cells are responsible for the removal and disposal of shed POS, a process vital for the renewal of photoreceptor membranes. Disruption of this process, as observed in the Royal College of Surgeons (RCS) rat [6], results in an accumulation of debris within the subretinal space, leading to degeneration of photoreceptors and eventual blindness [7,8].

RPE cells in vivo are distinct from many phagocytic cells, since they normally ingest only one type of particle: the POS [9]. Yet in culture, although RPE cells preferentially phagocytose POS, they can also bind and ingest a variety of substances, including POS, red blood cells, algae, bacteria, yeast, carbon particles, as well as inert particles such as polystyrene/latex beads [10-12]. The distinction between nonspecific and outer segment-specific phagocytosis is most readily observed in the RCS rat; although microvilli of the RCS rat RPE can envelop outer segments, they rarely ingest them [12,13]. Despite this, RPE cells from both RCS and normal rats are able to ingest polystyrene beads at the same rate in culture [12], suggesting that the RCS rat has a defect in POS-specific phagocytosis, rather than a defect in the general process of phagocytosis. For these reasons we believe that the phagocytosis of latex beads, which has been used previously to assess HESC-derived RPE phagocytosis [1,2], is an inappropriate assay with which to measure the functional capacity of these cells.

Some of the molecular mechanisms responsible for the phagocytosis of POS by RPE have been examined recently, and several proteins with proposed roles identified. Diurnal binding of POS to the RPE cell has been attributed to αVβ5 integrin, which is present at the apical surface of the RPE cell and selectively binds POS and not latex beads in vivo and in vitro [14-17]. CD36, a transmembrane glycoprotein, is described as functioning downstream and independently of αVβ5 integrin to control the rate of POS uptake [18]; when overexpressed, it induces the phagocytosis of POS by melanoma cells [19]. Other ligands thought to be involved in the regulation of outer segment binding include CD81, which interacts with and promotes particle binding to αVβ5, and milk fat globule-EGF8 (MFGE8), an αVβ5 integrin ligand required for diurnal phagocytosis of POS in vivo [20,21]. Ingestion of the outer segments is regulated through Mer Tyrosine Kinase (MERTK) [22]; mutations in this gene are responsible for defects in phagocytosis and the onset of retinitis pigmentosa in humans [23] and retinal dystrophy in the RCS rat [23,24]. Focal adhesion kinase (FAK) [25], which is responsible for the phosphorylation of MERTK, and MERTK ligands, such as growth arrest specific protein 6 (GAS6) [26-28] and protein S [29], are also thought to play important roles in the phagocytosis of outer segments, while cathepsin D, a major lysosomal enzyme present in RPE, is involved in the degradation of internalized POS [30].

Recently we have derived and characterized RPE cells from the Shef1 HESC line [4,31]. Here we examine the ability of these HESC-derived RPE to phagocytose POS isolated from the porcine retina and employ a novel in vitro assay to examine phagocytosis of outer segments from human retinal explants. Our results show that HESC-derived RPE cells express the molecules associated with phagocytosis and can phagocytose both outer segments and latex beads, but require MERTK for specific phagocytosis of outer segments. Furthermore, exposure to an ex vivo preparation of human retina leads to some morphological changes in the HESC-derived RPE layer, which may be indicative of cell maturation.

## Methods

### Cell culture

All tissue culture reagents, unless stated otherwise, were obtained from Invitrogen (Paisley, UK). The Shef1 HESC line was maintained in T25 flasks coated with 0.1% gelatine and seeded with mitomycin C inactivated mouse embryonic feeders (1.5×10^5^/T25) as described previously [32] using standard HESC-medium: knockout Dulbecco’s Modified Eagle’s Medium (DMEM) with 20% serum replacement, 1% non-essential amino acid solution, 1 mM L-Glutamine, 4 ng/ml human bFGF and 0.1 mM β-mercaptoethanol (Sigma-Aldrich, Gillingham, UK). Once removed from liquid nitrogen storage, the cells were maintained for 20 passages at 37 °C in 5% CO_2_ with medium changes every 2 days. At each passage cells were split 1:4 to maintain culture of undifferentiated colonies. Regular screening in the laboratory at Sheffield University confirmed that the cells used in this study were karyotypically normal. ARPE-19 cells were cultured as standard in T25 tissue culture flasks with medium containing DMEM/F-12 mix plus Glutamax with 1% penicillin-streptomycin and 10% FCS at 37 °C with 5% CO_2_. Cells were trypsinised and seeded onto 90 mm dishes. ARPE-19 cells were sampled when confluent at passage 22 for western blot and RT–PCR analysis.

### Generation of HESC-derived RPE

To generate HESC-derived RPE cells, we allowed Shef1 cultures to grow to confluence so that the borders of individual colonies fused together. It took approximately 1 week post-passage to achieve this condition, after which, medium changes (basic HESC-medium minus bFGF) were performed daily. Pigmented foci formed in the super-confluent HESC cultures 1–2 weeks following implementation of the daily medium change regime. These foci were then mechanically removed using the tip of a plastic pasteur pipette and microsurgical blades, before being placed onto 35 mm dishes pre-coated with Matrigel^TM^ Matrix (BD Biosciences, Oxford UK). The isolated pigmented foci (5–10 per dish) were cultured for a further 4 weeks in basic HESC-medium (minus bFGF) with medium changes every 2–3 days. The expansion of a subset of pigmented foci resulted in the formation of distinct patches of RPE monolayers within dishes that could be used in the phagocytosis assay or for immunocytochemistry. To analyze cell expression profiles by Western Blot and RT–PCR Shef1 cells were sampled before bFGF removal for the HESC-non pigmented control cells (HESC-NP). Cells were also sampled after the formation of pigmented foci in super-confluent culture for the HESC-derived pigmented RPE cells (HESC-P).

### Phagocytosis of POS isolated from porcine eyes

Retinas from freshly slaughtered porcine eyes (Cheale Meats Ltd., Brentwood, UK) were dissected free from the RPE under sterile conditions and POS isolated using a continuous sucrose gradient as previously described [33]. The POS pellet was resuspended in 1 ml, 10 mM sodium phosphate, and labeled with Alexa Fluor ® 488 Dye (Invitrogen) in 0.1 M sodium bicarbonate/5% sucrose in a light-tight microcentrifuge tube for 1 h at room temperature. Labeled POS were then washed and resuspended in standard HESC medium with 5% sucrose. POS were then seeded onto HESC-derived RPE monolayers grown on Matrigel™ Matrix at 1x10^7^ POS/ml Cells were incubated at 37 °C in 5% CO_2_ for various time intervals. They were treated for 10 min with trypan blue to remove external fluorescence then washed. Cells were fixed in cold 4% paraformaldehyde in 0.1 M PBS (138 mM NaCl, 3.89 mM KCl, 2.13 mM KH_2_PO_4_, 8.16 mM Na_2_HPO_4_) for 20 min at 4 °C. Finally, cells were washed 3 times in 0.1 M PBS for 5 min before they were processed for immunocytochemistry and confocal microscopy.

To examine the role of MERTK in phagocytosis, we incubated HESC-RPE cells with rabbit monoclonal antibody to 1:40 MERTK or 1:40 control rabbit IgG (both Abcam, Cambridge, UK) for 1 h. The medium was removed and replaced with medium containing fresh antibody and 1×10^7^ Alexa Fluor® labeled POS/ml or 1×10^7^ Fluorosphere polystyrene beads/ml (Invitrogen). Cells were then incubated for 5 h under normal culture conditions, treated with trypan blue and fixed.

### Phagocytosis of POS from human retina

HESC-derived RPE cells were expanded into monolayers on Matrigel™ Matrix coated 30 mm 0.45 μm Millicell culture plate inserts (Millipore, Watford, UK) placed inside a 6-well tissue culture dish. Human neural retina explants (surplus tissue from surgery at Moorfields Eye Hospital, with full local and national (COREC) ethical permission for research use) were collected in basic HESC-medium, and orientated on a 0.45 μm filter (as described in previous section) so that the POS surface was uppermost. The retina and its filter support were placed onto the HESC-RPE cells so that the outer segments were adjacent to the apical surface of cells. The filters were held together using a 12-mm plastic insert. Standard HESC-medium was added to the dish, and the insert weighted down using the dish lid. This artificial ex vivo system was incubated for up to 48 h at 37 °C in 5% CO_2_, after which the HESC-RPE plus neural retina composites were fixed for immunocytochemistry (with 4% paraformaldehyde in 0.1 M phosphate buffer) or for electron microscopy (with 1% paraformaldehyde and 3% glutaraldehyde in 0.08 M cacodylate buffer). Because of the limited supply of material, some experiments were repeated with porcine retina explants. After fixation HESC-RPE plus retina on filters were excised, washed in PBS and immersed in 30% sucrose in PBS overnight. They were then frozen in Tissue-Tek® O.C.T.™ Compound (VWR, Leicestershire, UK) with an acetone/dry ice slurry before sectioning onto charged glass slides (VWR). Processing for electron microscopy is described in the Electron Microscopy section.

### Immunocytochemistry

Sections of HESC-RPE or the dishes of HESC-RPE-fed outer segments were processed for immunocytochemistry as follows. After blocking for 30 min in 5% normal donkey serum (NDS; Stratech, Newmarket, UK) in PBS containing 0.3% Triton X-100 (PBS-TX) samples were incubated overnight in 1:100 anti-Na^+^/K^+^ ATPase (mouse monoclonal; Abcam), 10 μg/ml anti-αVβ5 Integrin (mouse monoclonal, Millipore), 1:50 anti-MERTK (rabbit monoclonal; Abcam) in PBS-TX plus 1% NDS. The following day, cells were washed in PBS before addition of FITC, TRITC, or Cy5-labeled antimouse or antirabbit secondary antibody (Stratech, diluted 1:200 with 2% NDS). Counterstaining with DAPI (4’6-diamindino-2-phenylindole dihydrochloride, Sigma), was followed by washing in PBS and mounting in Vectashield (Vector Laboratories Ltd., Peterborough, UK). Omission of the primary antibody revealed an absence of positive staining (data not shown). The uptake of fluorescent POS/polystyrene beads and immunocytochemical staining was analyzed using a Zeiss LSM 510 confocal microscope with LSM Image Browser software (Carl Zeiss Ltd. Welwyn Garden City, UK). Unless otherwise stated, images shown in figures are stacked confocal projections. To quantify levels of phagocytosis, we analyzed 5–6 distinct patches of HESC-RPE cells, originally derived from different pigmented foci, for each time point studied (ranging from 0.5 h to 20 h). In each case, images were taken blind and the total numbers of fluorescent POS or beads counted within the 60X magnification confocal field (150 μm×150 μm). Clumps of POS separated by more than 5 μm were counted as separate entities.

### Electron microscopy

After fixation in the paraformaldehyde and glutaraldehyde mixture, samples were fixed in 1% osmium tetroxide in cacodylate buffer. They were then dehydrated in a graded series of alcohols and epoxypropane before being embedded in Araldite CY212 resin (Agar Scientific, Standsted, UK). Ultrathin sections were stained with uranyl acetate and lead citrate and viewed with a Joel1010  electron microscope (Joel (UK) Ltd., Welwyn Garden City, UK).

### Western blot

Cells were placed on ice, washed with cold PBS. Cells were lysed on a tube rotator for 30 min at 4 °C in a lysis buffer that contained 10 mM HEPES, 1% Triton, 150 mM KCl, 1 mM PMSF, 10 ng/ml leupeptin, 1 mM DTT, 50 ng/ml aprotinin, 10 mM NaF, and 100 μM sodium vanadate). Samples were centrifuged at 12,000x g for 30 min at 4 °C and the supernatant recovered. The protein concentration for each supernatant was determined using Biorad Protein assay reagent (Biorad, Hemel Hempstead, UK) before dilution in sample buffer and denaturation at 95 °C for 5 min. Equal amounts of protein were separated on an SDS–PAGE gel and transferred onto Hybond PVDF membrane (GE Healthcare Life Sciences, Buckinghamshire UK). Membranes were incubated for 2 h in a blocking solution that contained 10% milk in PBS and 0.05% Tween-20. They were then incubated overnight at 4 °C with 1:500 anti-MERTK (rabbit monoclonal, Abcam), 0.1 μg/ml anti-FAK (rabbit polyclonal, Stratech), 0.4 μg/ml anti-αV integrin (mouse monoclonal; Stratech), and 0.4 μg/ml anti-β5 integrin (goat polyclonal; Stratech). Membranes were twice washed for 15 min in PBS and 0.05% Tween-20 before addition of secondary HRP-conjugated antibodies (Dako, Ely UK) in 10% milk, PBS, and 0.05% Tween-20. After 1 h the membranes were washed twice for15 min in PBS and 0.05% Tween-20 before they were incubated in LumiLight western blotting solution (Roche Products Ltd., Welwyn Garden City, UK). Proteins were detected with autoradiographic film. Blots were stripped using 8 M guanidium-HCl and reprobed using 1:1000 anti-GAPDH (goat polyclonal; Everest Biotech Ltd., Oxfordshire UK).

### Gene expression analysis

RNA was extracted from cells using TRIzol reagent (Invitrogen) according to the manufacturers protocol and contaminating DNA removed using RQ1 RNase-free DNase (Promega, Southampton, UK). First strand cDNA synthesis was performed from 3 μg total RNA using Superscript Reverse Transcriptase III (Invitrogen). Genomic control reactions for each RNA sample were included by omitting the reverse transcriptase, and a no template control, containing all the first strand synthesis reagents but lacking RNA template, was also prepared.

PCR was performed on the reverse transcription and control reactions using Go Taq Polymerase (Promega) with 0.2 μM of gene-specific primer (Eurofins MWG Operon, Ersberg, Germany) in a PCR Mastercyler® (Eppendorf, Cambridge UK). Briefly 1 μl cDNA was initially denatured at 95 °C for 2 min and amplified by 35 cycles of denaturation at 95 °C for 30 s, annealing at 3 °C lower than the melting temperature (Tm) for 30 s, and elongation at 72 °C for 30 s. PCR was completed with a final elongation step at 72 °C for 5 min. All products were resolved on 2% agarose gels with a 100 bp DNA ladder (Promega). Details of gene-specific primers can be found in Table 1.

**Table 1:** PCR primer sequences

**mRNA**	**Forward primer (5′−3′)**	**Reverse Primer (5′−3′)**	**Tm**	**Amplicon** **size**	**Accession No**
Itgav	CCTGTGCCTGTGTGGGTGAT	GGTGGCCGGACCCGTTTA	58	110	NM_002210
Itgb5	CGAGCGTGGGCACTGTCTCT	GCAGGCACTCGACGCAATCT	58	128	NM_002213
CD36	TGCAGCCTCATTTCCACCTT	CAAAGGCCTTGGATGGAAGA	54	151	NM_001001547
CD81	TCTGGAGCTGGGAGACAAGC	GGATGACCAGGCAGGTGAAG	58	163	NM_004356
Ctsd	AGCTGGGAGGCAAAGGCTAC	CCCTGTTGTTGTCACGGTCA	56	188	NM_001909
Cltc	CGTCACTGCACTCCATCCTG	ACCCACAGGGTCTCCACAGA	58	270	NM_004859
Clta	AGAGCCACCCTGTGGAAACA	GCTTCCCTCCCCTTCCTCTT	56	215	NM_001833
Cltb	AACGGTCCTGCTGATGGCTA	CACTCTGTGCCTGGGGTCTC	56	280	NM_001834
Fak	ATGTGACGGGCCTGGTGAAAGG	TGGGTGCTGGCTGGTAGGAG	58	160	AY323812
Gas6	GCGGAATCTGGTCATCAAGG	TCAGCCAGTTCCAGCTCCTC	56	199	NM_000820
MerTK	GTGAGGCAGCGTGCATGAAAG	GGGCTTTGGGATGCCTTGAG	58	95	NM_006343
Mfge8	CTTGGCTTCTCAGCCCCTTT	GTGAGGACTGGGGGTTAGGG	56	209	NM_005928
ProS1	CGGAAAATGGATTGCTGGAA	ACCAGAAACCAAGGCAAGCA	52	340	NM_000313
Tbp	GAACCACGGCACTGATTTTC	CCCCACCATATTCTGAATCT	52	157	NM_003194

Primers were designed to amplify human sequences for alphaV integrin (Itgav), β5 integrin (Itgb5), CD36 molecule (CD36), CD81 molecule (CD81), Cathepsin D (Ctsd), Clatherin - heavy chain (Cltc), and light chains, (Clta and Cltb), Focal Adhesion Kinase (Fak), Growth arrest specific gene 6 (Gas6), Mer Tyrosinse Kinase (MerTK), Milk fat globule-EGF factor 8 protein (Mfge8), Protein S (ProS1), and TATA box binding protein (Tbp).

## Results

### Phagocytosis of POS isolated from porcine retina

**Figure 1:**
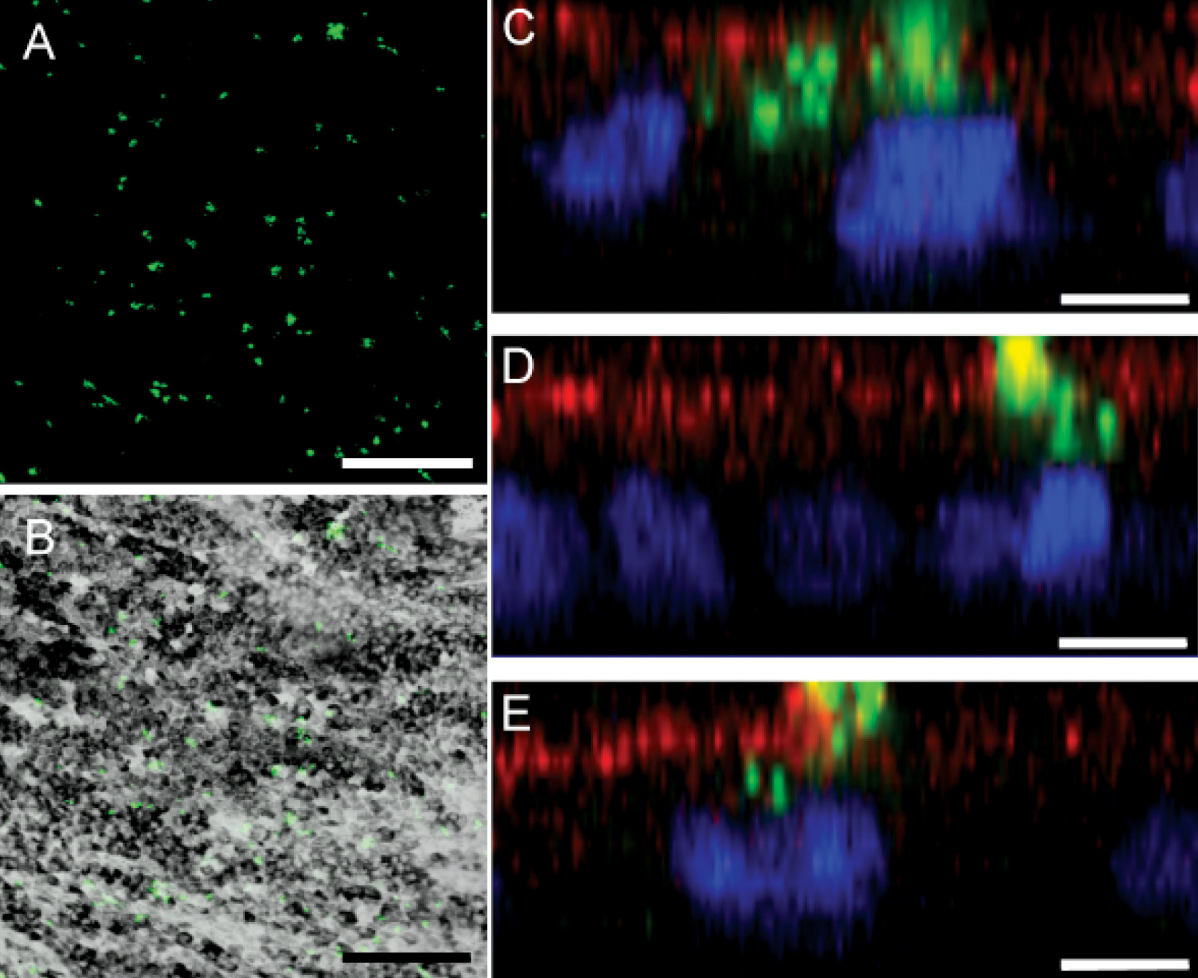
HESC-derived RPE cells internalize fluorescently labeled POS isolated from the porcine eye. **A**: The pigmented monolayer of HESC-derived RPE binds labeled POS (green). **B**: A Nomarski image showing pigmented HESC-derived RPE overlaid with Alexa Fluor® 488-labeled porcine POS. Evidence of internalization of outer segments is clear by 4 h. **C-D:** Alexa Fluor® 488-labeled porcine POS are associated with the apical surface of HESC-derived RPE (delineated by TRITC-labeled Na^+^/K^+^ ATPase immunostaining). **D-E:** POS are ingested by the cells and are located close to the HESC-derived RPE nuclei (DAPI, stained blue). Photomicrographs in **C-E** are single (y-axis) confocal optical slices (<0.8 μm) from 3 separate HESC-RPE samples. In **A-B**, scale bars equal 150 μm; in **C-E**, scale bars equal 10 μm.

**Figure 2:**
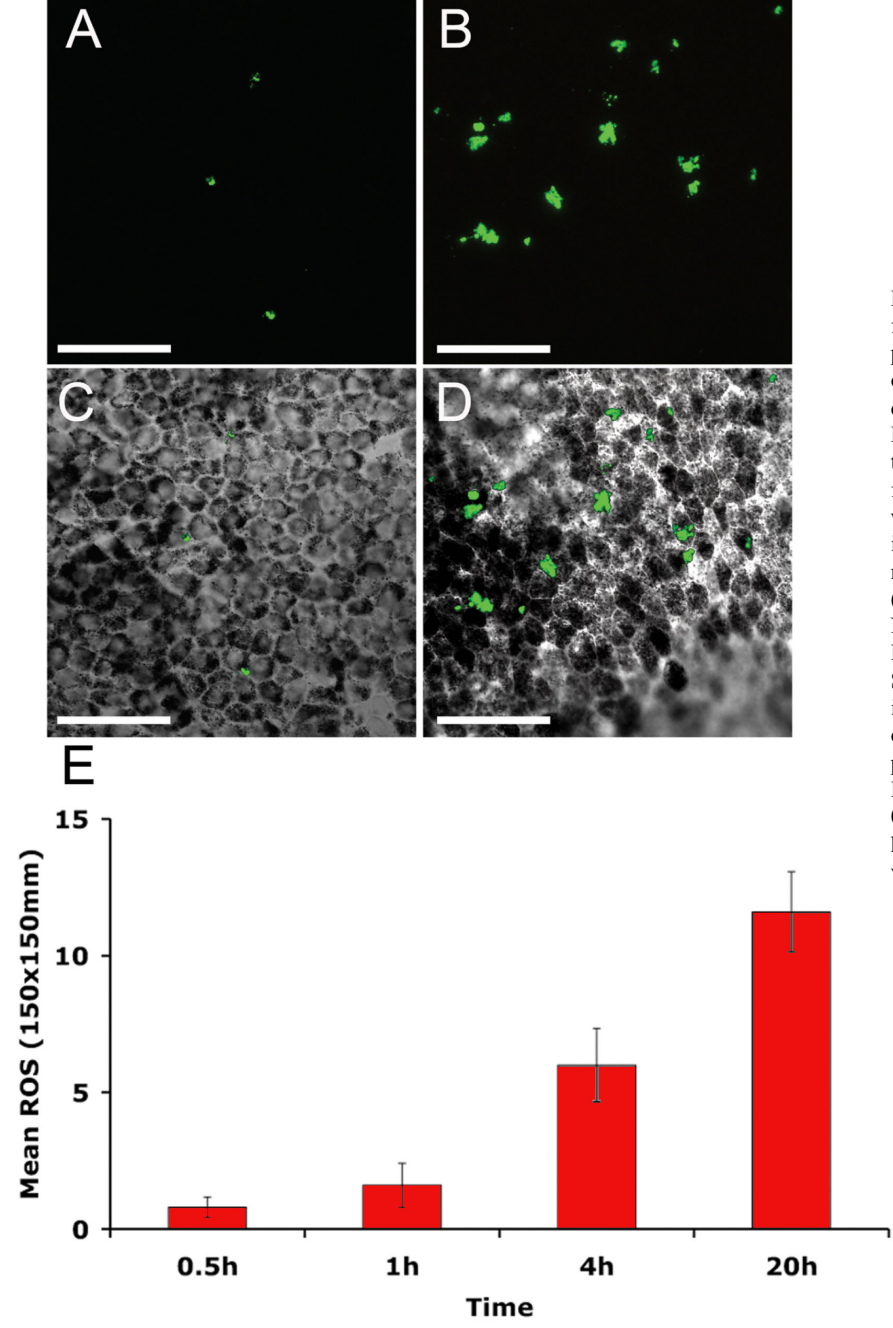
Increasing numbers of fluorescently labeled porcine POS are phagocytosed by HESC-derived RPE over time. A monolayer of HESC-derived RPE cells was exposed to Alexa Fluor® 488-labeled porcine POS, treated with trypan blue to remove fluorescent non-internalized POS, washed, fixed, and processed for immunocytochemistry. Representative micrographs are shown from (**A**) 1 h and (**B**) 20 h together with respective Nomarski images (**C** and **D**). Alexa Fluor® 488-labeled POS are in green. Scale bars equal 50 μm. **E** Increased internalization of POS was observed over time. Data shown are mean±SEM p<0.001, One-way ANOVA with Bonferroni multicomparison test, (n=5); 0.5 h versus 4 h p<0.05, 0.5 h versus 20 h p<0.001, 1 h versus 20 h p<0.001, 4 h versus 20 h p<0.05.

To address the ability of HESC-derived RPE cells to phagocytose outer segments we exposed cells to fluorescently labeled POS isolated from porcine retina in culture. After 4 h there was uniform uptake of POS across the pigmented HESC-derived RPE cell monolayer (Figure 1A,B). To ensure that labeled POS were internalized we examined y-axis sections through cells. Confocal microscopy imaging of cells exposed to outer segments and immunostained for the RPE cell apical surface marker Na^+^/K^+^ ATPase demonstrated that these cells actively internalize outer segments. Fluorescently labeled outer segments are observed within the apical region of the cell (Figure 1C,D), and inside the cell, proximal to the DAPI stained nucleus (Figure 1E). Labeled POS were observed in RPE cells at all time points examined (0.5 h–20 h). Representative images from 1 h and 20 h are shown in Figures 2A,B alongside respective Nomarski images (Figure 2C,D). Increasing numbers of outer segments are internalized by HESC-derived RPE over time (Figure 2E, p<0.001, One-way ANOVA).

### Role of MERTK in HESC-RPE phagocytosis

**Figure 3:**
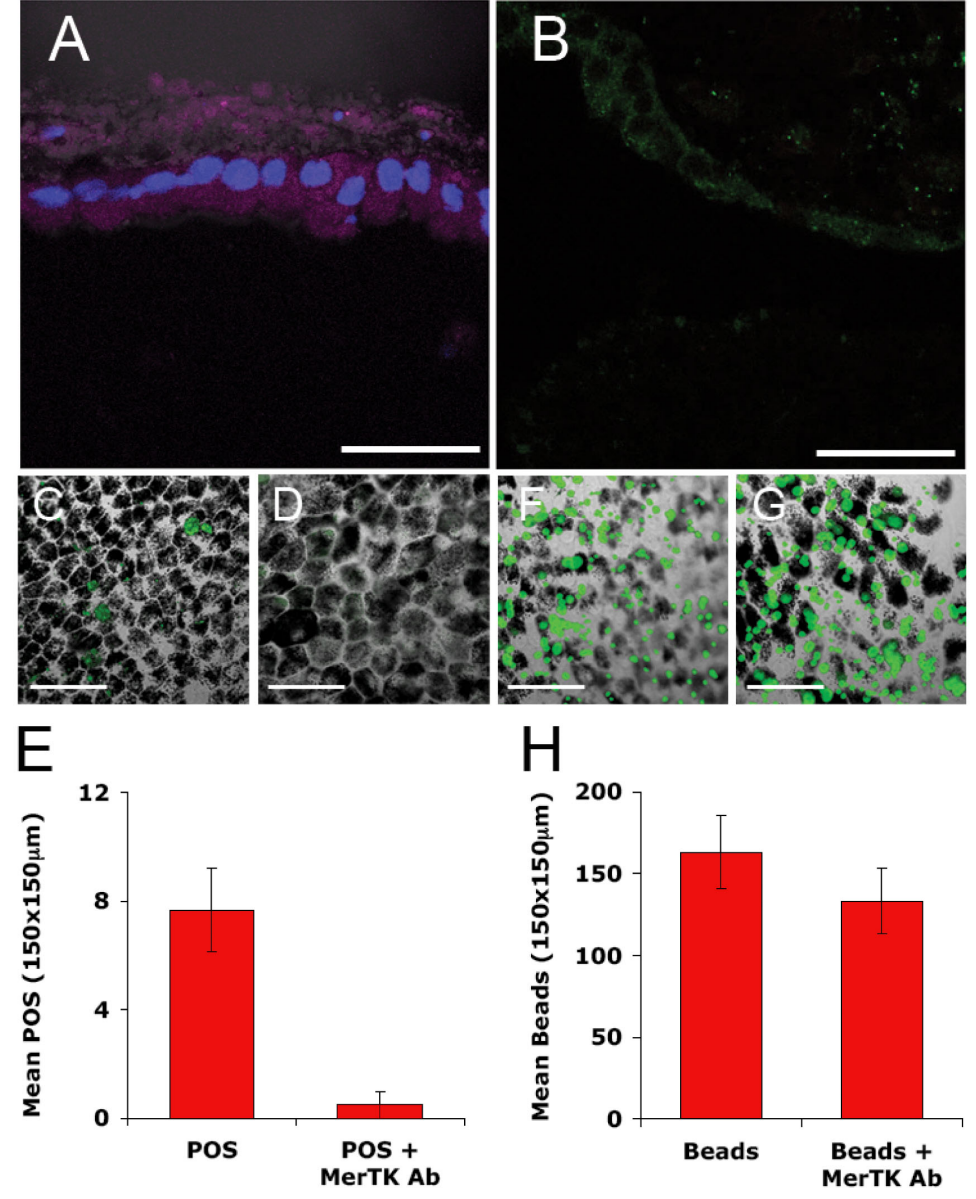
HESC-RPE cells require MERTK for the internalization of POS. HESC-RPE cells express the phagocytic proteins (**A**) MERTK and (**B**) αVβ5 integrin. To determine the importance of MERTK in the phagocytosis of POS, we treated HESC-RPE cells with control IgG (**C** and **F**) or MERTK antibody (**D** and **G**) for 1 h before exposure to Alexa Fluor® 488-labeled porcine POS (**C** and **D**) or fluorescent polystyrene beads (**F** and **G**) for 5 h. Cells were treated with trypan blue, washed, and fixed. The number of internalized POS (**E**) and fluorescent beads (**H**) was then quantified per field view (150 μm  × 150 μm). Data shown are mean±SEM (n=6). Pre-incubation with MERTK antibody had a significant effect on the number of outer segments ingested by HESC-RPE cells (p<0.01, Student *t*-test, n=6). There was no effect of MERTK antibody on the ingestion of polystyrene beads by the cells (p>0.05, Student *t*-test, n=6). Photomicrographs are single confocal optical slices merged with Nomarski image (<0.8 μm). Scale bars equal 50 µm. MERTK is labeled with Cy5 and counterstained with DAPI, αVβ5 is labeled with FITC.

HESC-RPE cells expressed two key molecules known to be involved in phagocytosis by RPE cells: MERTK and αVβ5 integrin (Figure 3A,B respectively). MERTK is expressed throughout the cytoplasm and in the apical microvilli while αVβ5 integrin is expressed toward the cell surface (Figure 3A,B). To examine the role of MERTK in phagocytosis by HESC-RPE cells, we used a MERTK antibody to block receptor function. HESC-RPE cells exposed to MERTK antibodies showed a significant reduction in the number of POS ingested over a 5 h period compared to control cells (Figure 3C-E, p<0.01, Student *t*-test). Under the same conditions the number of latex beads ingested by HESC-RPE cells remained unaffected by incubation with MERTK antibodies (Figure 3F-H, p>0.05, Student *t*-test).

### HESC-derived RPE cells express molecules associated with phagocytosis of POS

**Figure 4:**
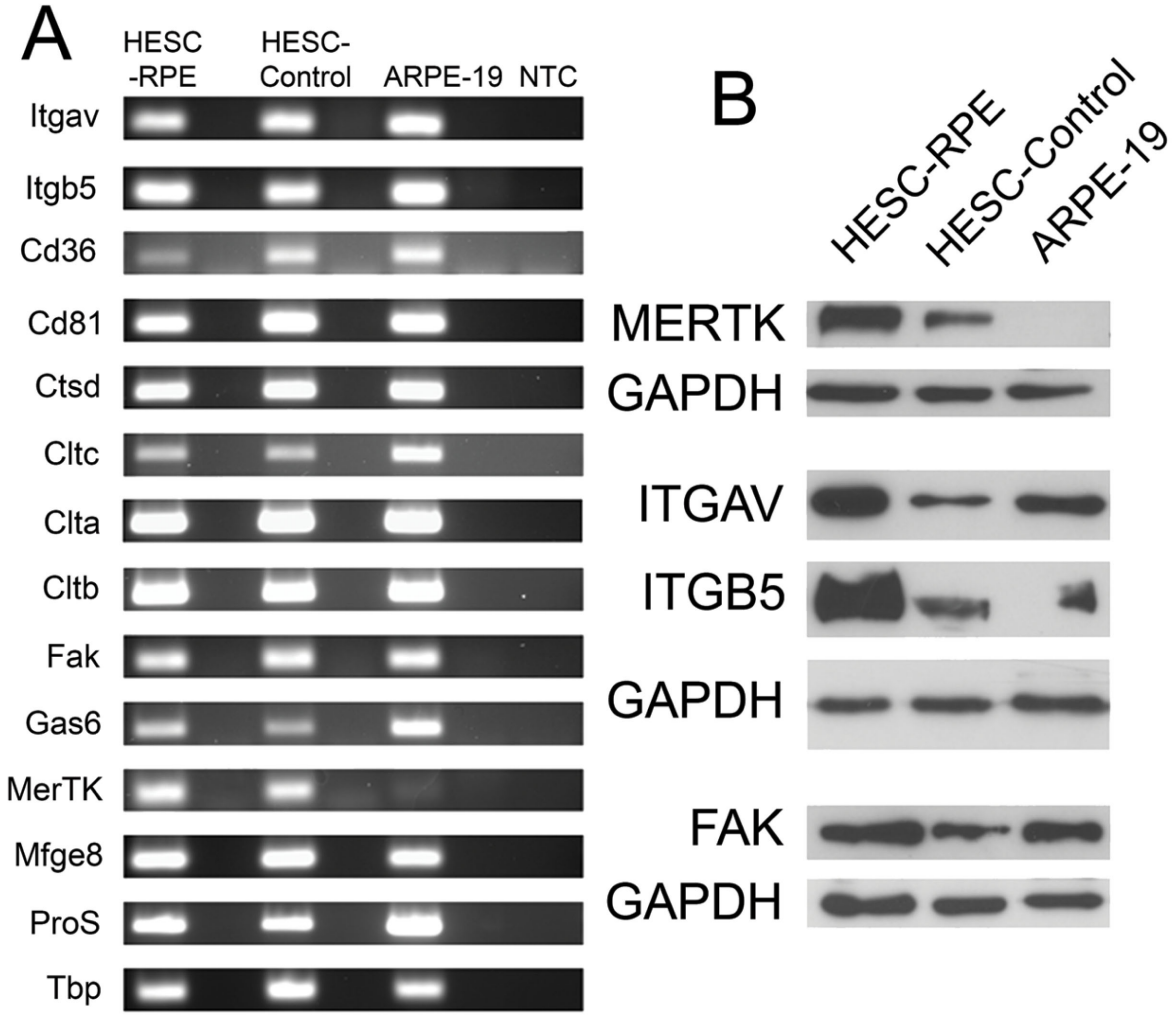
HESC-derived RPE cells express molecules required for POS specific phagocytosis. **A:** RT–PCR analysis of gene expression in HESC-derived RPE cells (HESC-RPE), control nonpigmented HESC (HESC-Control) and the human RPE cell line ARPE-19. cDNA was synthesized from 3 μg of RNA in a 20 μl reaction, from which 1 μl was used for PCR amplification. Lanes to the right of each sample are genomic DNA control reactions, which lacked the reverse transcriptase during cDNA synthesis. NTC is a no RNA template sample to control for non-specific amplification. All PCR reaction products were resolved by agarose gel electrophoresis. PCR primer sequences, amplicon size, and melting temperatures are described in Table 1. *Tbp* was amplified in all samples as a housekeeping control gene. **B:** western blot analysis of protein expression in HESC-P, HESC-NP and ARPE-19 cells. Equal amounts of protein were resolved on an SDS–PAGE gel, transferred onto membranes, and hybridized with antibodies for MERTK (180 kDa), focal adhesion kinase (FAK, 125 kDa), αV integrin (125–135 kDa), and β5 integrin (100 kDa). Membranes were stripped and re-probed with GAPDH (36 kDa) as a loading control.

We next investigated the expression of molecules proposed to be involved in the POS specific pathway in HESC-RPE cells using PCR and western blot (Figure 4). HESC-P, HESC-NP, and ARPE-19 cells express many of the mRNAs of molecules associated with the binding of outer segments including *Itgav* and *Intb5*, *Cd36*, *Cd81*, and *Mfge8* (Figure 4). Cells also express molecules implicated in the internalization and degradation of outer segments: *Gas6*, *Fak*, *Pros1*, *Ctsd,* and *Clt-a*, *-b*, and *-c*. The gene required by RPE cells for POS specific internalisation, *Mertk*, is expressed in HESC-P and HESC-NP cells; however the mRNA for this gene could not be amplified from ARPE-19 cells. This finding was confirmed at the protein level using western blotting (Figure 4B). MERTK was expressed in HESC-P and, at lower levels in HESC-NP, but was absent in ARPE-19 cells. FAK, ITGAV, and ITGB5 were detected in all cells examined. Full-length *Mertk* cDNA was sequenced from the HESC-derived RPE cells and found to be normal (data not shown).

### Phagocytosis of human POS in a novel in vitro assay

**Figure 5:**
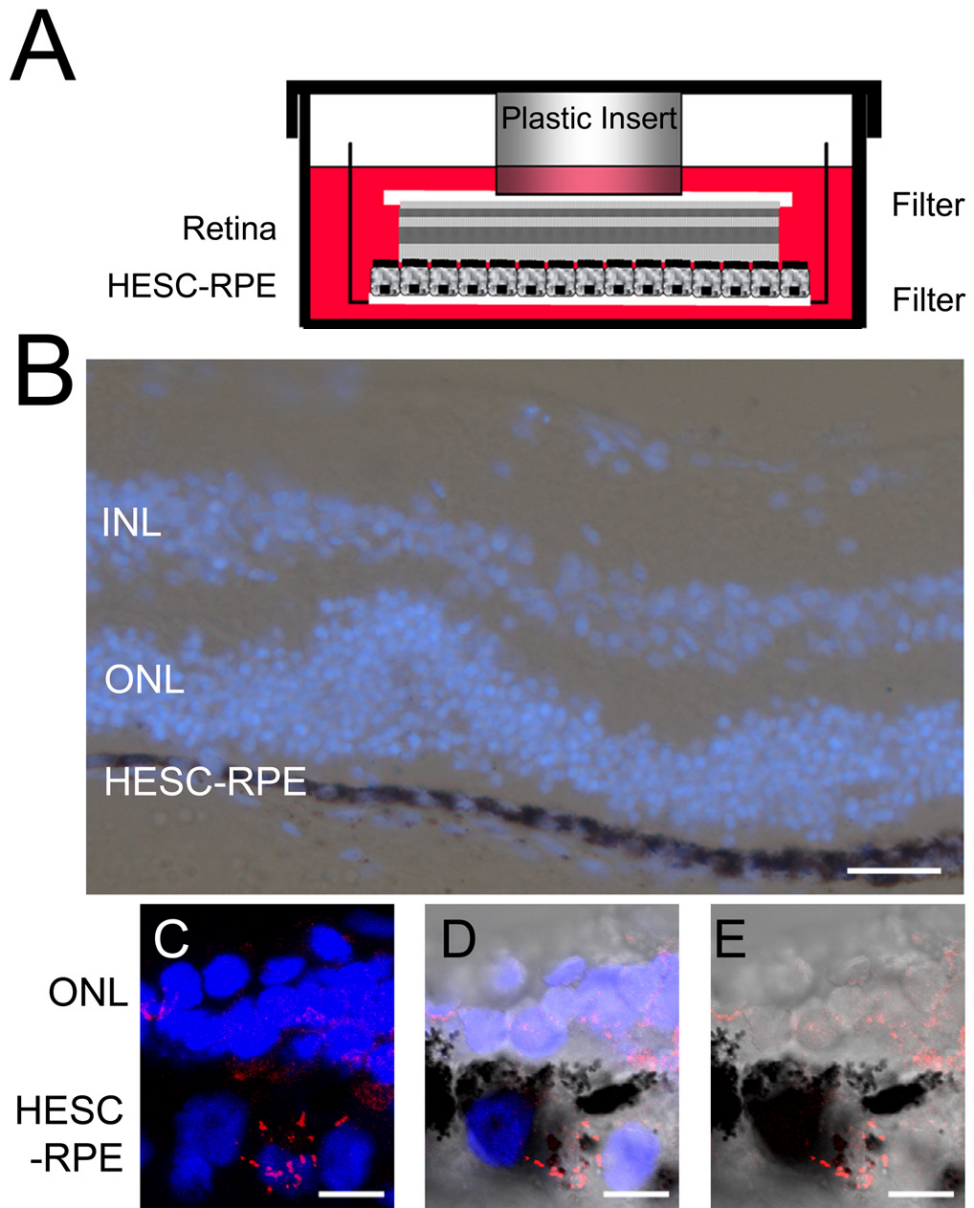
A novel in vitro assay to assess phagocytic activity in HESC-derived RPE cells. **A:** A schematic illustration of the novel in vitro retinal explant model system used to analyze phagocytosis. HESC-derived RPE cells were cultured on a Matrigel™-coated filter and exposed to the photoreceptor side of human or pig retina placed on a filter. The RPE-retina “sandwich” was cultured in HESC medium and held together by a plastic insert weighted down by the 6-well plate lid. **B:** A Nomarski image of HESC-derived RPE exposed to a porcine retina explant for 48 h in vitro. The nuclei of cells are stained with DAPI (blue). The outer nuclear layer (ONL), indicating the rod and cone nuclei, and inner nuclear layer (INL), specifying the amacrine, bipolar, and horizontal cell layers are labeled. **C-E**: Immunohistochemical staining for rhodopsin in HESC-derived RPE cells exposed to porcine retina for 48 h in the novel retinal explant system. **C** is a confocal projection and **D-E** are single confocal slices (<0.8 μm) merged with the Nomarski image. The rhodopsin staining (TRITC labeled) observed within pigmented HESC-derived RPE cells is indicative of POS phagocytosis. The nucleus is stained with DAPI (blue). Scale bar in **B** equals 50 µm; scale bar in **C**, **D**, and **E** equals 20 µm.

To assess the potential of HESC-derived RPE to phagocytose outer segments from human retina we designed a novel in vitro assay. Using human retinal tissue, supplied by Moorfields Eye Hospital, we constructed an artificial ex vivo RPE and neural retina model system. Monolayers of HESC-derived RPE cells, cultured on Matrigel™-coated filters, were exposed to the photoreceptor cell layer of retina tissue explants. Contact between the RPE and photoreceptor outer segments was maintained by holding the layers together within the tissue culture dish with a plastic insert weighed down by the dish lid. A schematic of this system is shown in Figure 5A. Using porcine retina explants for initial studies, we could see that HESC-derived RPE cell and retina tissue morphology was maintained within this system after 48 h co-culture (Figure 5B). In this preparation we also showed that rhodopsin, a molecule found in abundance in rod outer segments, was internalized by HESC-RPE cells (Figure 5C-E). The confocal projection and single confocal optical slices demonstrated that rhodopsin was localized within the pigmented cells after exposure to the outer segment surface of a porcine retina explant.

**Figure 6:**
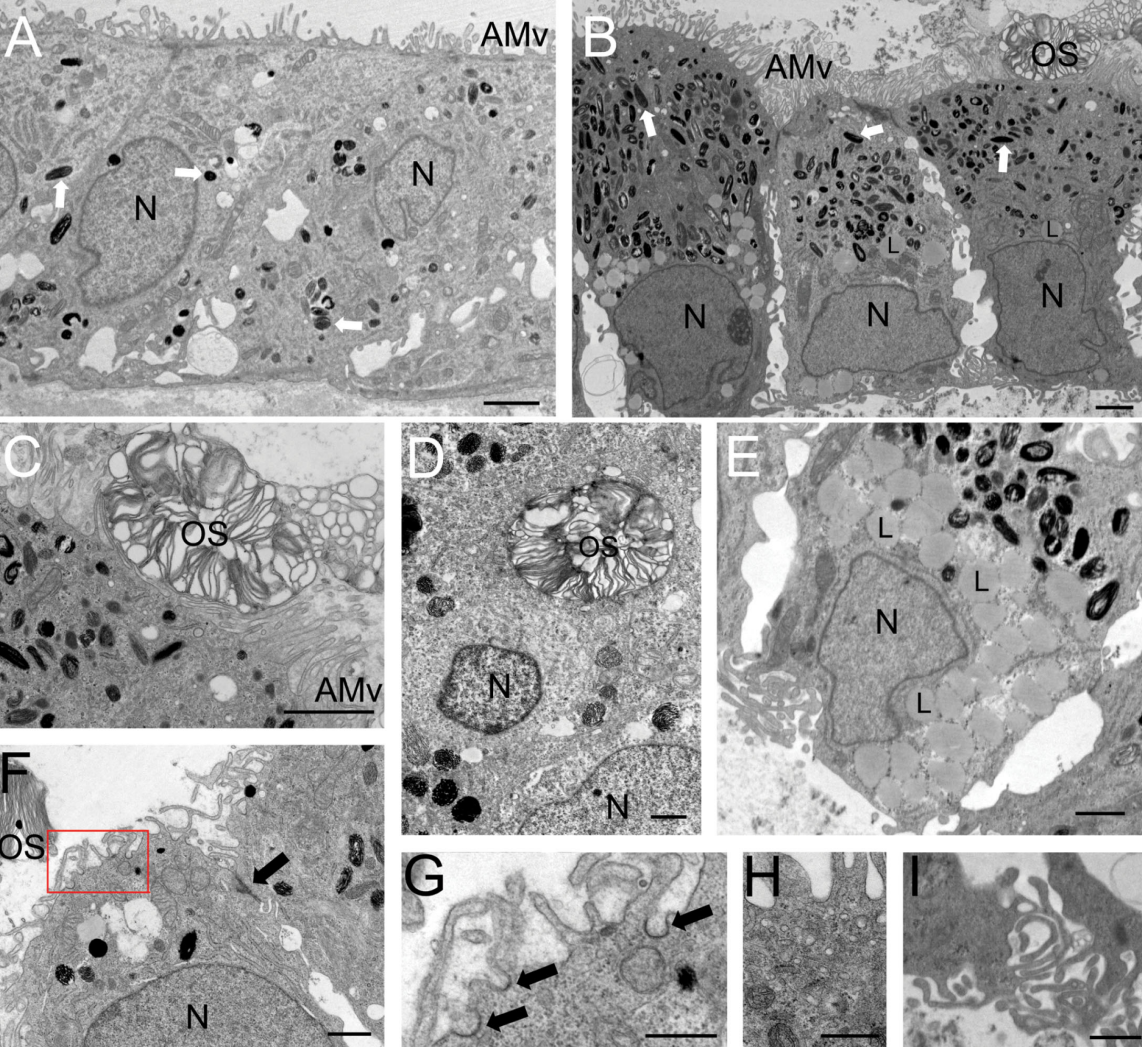
Electron microscopy of HESC-derived RPE cells after exposure to human retinal explant in vitro. **A:** Control HESC-derived RPE cells, which were not exposed to human retina, have apical microvilli (AMv) and contain melanin granules (white arrows). **B:** HESC-derived RPE exposed to the photoreceptor surface of human retina for 48 h appear more mature. Note the close association of the RPE apical microvilli with the photoreceptor outer segment (OS), the abundance of pigmented melanin granules within the apical region of the cell, the nucleus (N), and the numerous lipid deposits (L) located toward the basal portion of the cell. **C:** Apical microvilli surround a human photoreceptor outer segment. **D:** An outer segment is engulfed by the HESC-derived RPE cell. **E:** A high magnification image of lipid deposits (L) observed in the basal portion of a HESC-derived RPE after exposure to human retina. The formation of lipid deposits is indicative of the end stages of phagocytosis. Several features associated with RPE cell function are present in the cells including (**F**) tight junctions (black arrow) and coated pits (red box, indicated with arrows at higher magnification in **G**). Cells also contain a high number of coated vesicles within the apical portion of the cell (**H**) and develop basal end feet and infolding of the basal membrane (**I**). Scale bars equal 2 μm in **A**-**C**, 1 μm in **D**-**F**,**I**, and 500 nm in **G**,**H**.

HESC-derived RPE were exposed to small pieces of human retina explant in our model system for up to 48 h. As a control, HESC-derived RPE were cultured without the explant for the same period before analysis by electron microscopy. Control HESC-derived RPE cells, not exposed to human retina, closely resemble RPE cells; microvilli were present at the apical surface and melanin pigment granules were evident throughout the cytoplasm of the cell (Figure 6A). HESC-derived RPE cells exposed to human retina tissue readily bound POS (Figure 6B). Microvilli on the apical surface of the cell extended around the POS (Figure 6C), leading to its internalization (Figure 6D). Unlike the control HESC-RPE, the HESC-RPE in contact with retina had numerous lipid inclusions usually within the basal portion of the cell, close to the nucleus (Figure 6B,E). Similar results were obtained for HESC-RPE co-cultured with porcine retina (data not shown). Co-culture with human retina in this preparation led to alterations in the morphology of the HESC-derived RPE cells, normally associated with maturation. Under these conditions, the increased number and length of apical microvilli were accompanied by an increase in the number of melanin granules and premelanosomes, and a greater degree of cell polarization; pigment granules were concentrated toward the apical surface (Figure 6B). Coated pits (Figure 6F, and shown at high magnification in Figure 6G), coated vesicles within the cytoplasm of the cell (Figure 6H), and the presence of more pronounced infolding in the basement membrane (Figure 6I) were also evident in the HESC-derived RPE exposed to human retina.

## Discussion

AMD is a condition associated with the progressive dysfunction of RPE cells, leading to the death of photoreceptors and eventual central vision loss. The RPE is a monolayer of cells, which underlies the retina, and as such, it offers an accessible target site for treatment of AMD. Proof of concept for the use of human cells in grafting to preserve visual function has been demonstrated in studies involving transplantation of various cell types into the subretinal space of dystrophic animals [31], including RPE [3,34], and in human RPE transplantation trials [35,36]. The pluripotent potential of embryonic stem cells makes them an ideal source of material for use in regenerative medicine and cellular therapy. Several papers have described the potential of HESC to differentiate into RPE cells in vitro [1-4,31]. In culture, stem cells differentiate to form monolayers of pigmented cobblestone-like cells that express markers specific to RPE cells including bestrophin, CRALBP, and RPE65 [1,3,4]. Cells are also polarized, with microvilli observed in the apical portion of the cell and the formation of a basement membrane. Although functional analysis of HESC-derived RPE demonstrates that they are capable of phagocytosing latex beads [1,2], in vivo, RPE cells will normally phagocytose only POS. Despite the characterization of HESC-derived RPE, previous studies have not addressed the phagocytosis of POS.

The phagocytosis of POS is crucial for the survival of photoreceptor cells; dysfunction in this process leads to clinical disorders characterized by the degeneration of the retina and eventual blindness. Here, using confocal microscopy, we showed that monolayers of HESC-derived RPE cells are able to bind and internalize fluorescently labeled porcine POS and outer segments from a pig retina explant, as indicated by the incorporation of rhodopsin-positive POS fragments within the cells. Over time there was a continual increase in the number of fluorescently labeled POS associated with HESC-derived RPE cells. No saturation of ingestion was observed over the 20 h period of exposure, which is in agreement with studies of phagocytosis in primary human RPE cell cultures [37].

Of specific interest, when assessing cells for use in human clinical retinal therapies, is their ability to phagocytose human POS. Due to the limited supply of viable retina tissue, we designed a novel assay, whereby fresh human retina explants were co-cultured with HESC-derived RPE cells in an ex vivo model system. Using electron microscopy, we showed phagocytosis of POS from human retinal tissue by the HESC-derived RPE. We have been able to observe various stages of phagocytosis including the interaction of the POS with apical microvilli and their engulfment into the cell. After 48 h in co-culture with the retina, numerous lipid inclusions were observed within the basal portions of cells, indicative of terminal stages of segment digestion. In future, it will be important to examine phagocytosis of POS by RPE cells after transplantation into animal models of retinal dystrophy to demonstrate that photoreceptor survival is due to RPE-specific functions by donor cells rather than to growth factor-mediated rescue.

Phagocytosis of POS by RPE cells is a complex process and as yet, the full mechanisms responsible remain unclear. Several genes implicated in the three stages of RPE phagocytosis (recognition, ingestion, and digestion) are expressed in HESC-derived RPE and control cells, including *αv integrin* and *β5 integrin*, *Cd36* and *Cd81*, *cathepsin D*, the *clathrin* heavy and light chains, *focal adhesion kinase*, *Gas6*, *MerTK*, *Mfge8*, and *ProS*. We have also confirmed the expression of αVβ5, MERTK, and FAK proteins by the cells using western blot and immunocytochemistry. Control HESC also express this range of molecules which indicates that, once confluent, non-pigmented precursors expressing RPE cell markers are present within the population of cells before the removal of bFGF. These precursors can be identified using the antibody Pmel17, which stains pre-melanosomes [4]. Of particular interest to us is the expression of full-length (180 kDa) MERTK in HESC-derived RPE cells, which we have demonstrated to be crucial to the specific phagocytosis of outer segments. Using an antibody against MERTK to block function inhibits the uptake of outer segments, but has no effect on the ingestion of latex beads, suggesting that the specific phagocytosis of outer segments by HESC-RPE cells is mediated through the MERTK receptor, while nonspecific phagocytosis (i.e., polystyrene beads) is mediated through a separate mechanism.

It is worth noting that of all the molecules examined, MERTK was the only one not detected in our stock of ARPE-19 cells at the mRNA or protein level under the described culture conditions. Mutations in MERTK, which underlie the dystrophic phenotype observed in the RCS rat [22,24,38] and some human forms of retinitis pigmentosa [23,39], revealed the importance of this protein in the maintenance of a healthy retina. It is known that the gene expression profile of HESC-derived RPE more closely resembles that of freshly isolated RPE, when compared with human RPE cell lines [1]. Accordingly, this also appears to be true at the protein level. Even though ARPE-19 cell transplantation has beneficial effects in rats [34], the complete absence of MERTK and other molecules critical to RPE cell function [1-4,40] suggest that caution should be taken when investigating established RPE cell lines for use in human transplantation therapies. However ARPE-19 cells may prove to be a useful model system for unraveling the effects of the absence of MERTK in a human cell line.

The morphological appearance of HESC-derived RPE cells resembles that of the RPE in vivo before co-culture. However it is interesting to note that, after 48 h co-culture with human retina, cells appeared more mature. Structural changes observed in the cells are reminiscent of those observed in the rat RPE postpartum, with increases in the length of apical microvilli and the appearance of basal membrane infolding [5,41]. We observed an increase in the number of melanosomes, and in particular the number of late stage 3–4 melanosomes within the apical region of the cells. These findings concur with studies in vivo, suggesting that injections of ROS can induce melanogenesis in adult rat RPE cells over 5 days [42]. The shorter time period of melanogenesis observed in these cells may well reflect the immature state of HESC-derived RPE, having never been exposed to a POS. Other changes within the cells suggests that exposure to POS/retina may contribute to the functional maturity of the RPE. The presence of coated pits, and the emergence of coated vesicles and basement membrane infoldings are all signs of increased functional potential, suggesting the HESC-derived RPE are regulating cellular transport via the endocytosis of substances at the apical surface and the exocytosis of substances at the basal membrane. Tight junctions regulate the diffusion of substances across epithelial cell monolayers and function in the RPE as an integral component of the blood:retina barrier. The presence of tight junctions and their associated proteins [4] in HESC-derived RPE provides further evidence to suggest that these cells possess critical barrier properties.

One of the inherent problems of cell culture is the removal of cells from their original tissue source. Although adult RPE cells grow well in culture, repeated passage of cells derived from human RPE tissue results in the differentiation of cells away from their original phenotype, typified by high levels of proliferation, changes in gene and protein expression, decreased levels of pigmentation, and loss of structural characteristics [4,40,43-45]. Together these data demonstrate how critical the microenvironment is in the maintenance/restoration of RPE cell lineage. Previous studies have demonstrated that manipulation of the microenvironment has profound effects on RPE cells, specifically, the introduction of POS can induce changes in gene expression [44,46], tyrosinase biosynthesis and activity [47], and, pertinent to data presented here, increased levels of melanogenesis [42]. It is clear that RPE cells can differentiate from HESC in culture [1-4,48]; however, full structural and functional maturation may require in vivo signals from the retina. These findings also have implications for the differentiation of stem cells for clinical therapies. Perhaps only when cells are transplanted into the appropriate environment will full differentiation and functional maturation be observed.

In conclusion, we have demonstrated that HESC-derived RPE have the necessary molecular profile for the specific phagocytosis of outer segments, and require MERTK to mediate this process. Using a novel in vitro system, we have shown that HESC-derived RPE cells can phagocytose human outer segments, since there is evidence of engulfment and end stage lipid accumulations. Additionally, co-culture with retinal tissue may contribute to structural changes in RPE cells associated with maturation and increased functionality.
